# Healthcare costs and resource use in advanced breast cancer at the end of life: a register study

**DOI:** 10.2340/1651-226X.2026.44970

**Published:** 2026-02-06

**Authors:** Giovanni Galvis Rojas, Peter Strang, Torbjörn Schultz, Lars-Åke Levin

**Affiliations:** a Department of Health, Medicine and Caring Sciences, Linköping University, Linköping, Sweden; b Research and Development Unit, Stockholm Sjukhem Foundation, Stockholm, Sweden; c Department of Oncology-Pathology, Karolinska Institutet, Stockholm, Sweden

**Keywords:** Advanced breast cancer, healthcare costs, generalized linear models, palliative care, systemic treatment

## Abstract

**Background and purpose:**

Advanced breast cancer (ABC) involves substantial end-of-life (EOL) healthcare use and costs. Understanding cost drivers can inform care delivery and resource allocation.

**Patient/material and methods:**

We conducted a retrospective, population-based study of individuals (*n* = 1,437) who died with breast cancer in the Stockholm Region (2015–2023). Healthcare utilization and costs during the last 12 months of life were obtained from the Stockholm Regional Healthcare Data Repository (VAL) and estimated using the Region Stockholm cost model. Variables included age, sex, socioeconomic status (Mosaic), Charlson Comorbidity Index, Hospital Frailty Risk Score (HFRS), systemic therapy, and place of death. Descriptive statistics and generalized linear models assessed cost associations.

**Results:**

Total costs rose toward EOL, increasing 140% in the final 3 months versus the prior quarter. Hospitalizations and specialized palliative care drove costs, while outpatient visits declined. Younger age (18–69 years), high frailty (HFRS > 15), and systemic therapy were independently associated with higher costs. Hospital death was associated with lower expenditures than dying elsewhere (rate ratio [RR]: 0.84, 95% confidence interval [CI]: 0.78–0.91). The top 5% of cost users were mainly younger, frail patients receiving systemic therapy.

**Interpretation:**

ABC-related costs escalate in the final year of life, driven by hospitalizations, palliative care, and systemic therapies. Younger, frailer patients incur higher costs, while those dying in hospital settings are associated with lower costs. Early palliative integration and frailty-based risk stratification were associated with distinct patterns of healthcare utilization and costs toward the EOL.

## Introduction

Breast cancer (BC) remains one of the most commonly diagnosed malignancies among women globally [1]. The increasing incidence and prevalence of cancer, coupled with demographic shifts such as population aging and advancements in both diagnostic and therapeutic modalities, have led to a substantial rise in healthcare expenditures. In Europe, the total economic burden of cancer was estimated at approximately €199 billion, with healthcare-related costs nearly doubling from €52 billion in 1995 to €103 billion in 2018, as reported by Hofmarcher et al. [2]. Cancer care costs vary considerably by disease stage. For instance, Sun et al. demonstrated that the average cost of treating stage IV cancer was 109% higher than that of stage I, primarily due to the adoption of more complex and resource-intensive treatment strategies [3].

Healthcare expenditures are seen also throughout the disease trajectory, with peaks typically occurring during the initial diagnosis phase and the terminal stage, with emergency hospitalizations constituting a primary contributor to increased end-of-life costs (EOL) [4]. A stage-specific cost analysis of BC in Germany further corroborated these findings, indicating that the treatment costs for stage IV disease are approximately 95% higher than those for stage I, primarily due to expenditures associated with EOL care [5].

Bentur et al. found that, in Israel, the escalation in health care expenditure during the final 6 months of life is predominantly attributable to inpatient admissions, which account for 68% of the total costs. The remaining expenses are primarily associated with home hospice care and oncology day care services [6].

Aggressive interventions near the EOL, such as chemotherapy and immunotherapy, are frequently linked to a decline in patients’ performance status (PS). In addition, reliance on emergency department services and intensive care unit (ICU) admissions not only generates significant financial burdens on healthcare systems but also exposes patients to increased stress and discomfort, often unnecessarily [7, 8]. For this reason, the ESMO clinical guidelines for managing adult patients at EOL recommend avoiding the use of chemotherapy and immunotherapy during the last weeks of life, underscoring the importance of prioritizing patient comfort and quality of life over aggressive interventions at this stage [9].

Although there is growing interest in optimizing rather than maximizing care at the EOL, data on healthcare utilization and cost drivers in advanced breast cancer (ABC) remain limited. Several studies aggregate data across all solid tumors or focus primarily on therapy-related costs, with relatively less emphasis on the full range of healthcare services used during the final months of life [10]. For instance, a retrospective analysis of patients diagnosed with metastatic BC demonstrated that, although a significant proportion received inpatient palliative care, only approximately one-third were referred to hospice services during the final months of life. These findings highlight potential shortcomings in the coordination of care and the allocation of appropriate resources for EOL management [11].

Recent population-based studies from the Stockholm Region have demonstrated that receipt of specialized palliative care is associated with differences in healthcare utilization during the last phase of life among patients with cancer [12]. Understanding these patterns is essential not only for improving the quality of EOL care but also for ensuring the efficient allocation of healthcare resources. Strang et al. highlighted that younger age and increased frailty risk (an often-underrecognized factor) are associated with higher EOL expenditures. Moreover, individuals diagnosed with advanced-stage hematologic malignancies are observed to incur substantially higher EOL health care expenditures compared to those with solid tumors or nonmalignant conditions. Notably, significant heterogeneity in EOL spending is also evident across various solid tumor types, including BC. Conversely, socio-economic indicators at the area level, such as categorization by Mosaic groups and gender, appear to exert minimal influence on the overall cost burden associated with EOL care in these populations [13]. A Swedish study has reported variation in palliative care needs by sex and clinical characteristics among cancer patients prior to specialist palliative care referral [14].

Understanding these patterns is essential not only for improving the quality of EOL care but also for ensuring the efficient allocation of healthcare resources.

This study systematically evaluates healthcare resource utilization and costs during the final year of life in patients with ABC. It also examines associations with age, sex, socio-economic status at area level (Mosaic), comorbidity, frailty, systemic oncological therapies, and death in emergency hospital settings.

## Patients/material and methods

In alignment with the STROBE (Strengthening the Reporting of Observational Studies in Epidemiology) recommendations, the methods, results, and discussion sections have been structured, where relevant, to ensure comprehensive and standardized reporting of observational study findings [15, 16].

### Study design

This study is a retrospective, registry-based analysis designed to examine healthcare expenditures during the final year of life among patients with BC in the Stockholm Region. The analysis utilizes real-world data obtained from the Stockholm Regional Healthcare Data Repository (VAL), which captures nearly all inpatient and outpatient healthcare encounters within the region. The available data include information, for example, on outpatient consultations, durations of inpatient admissions, and diagnostic codes based on the ICD-10 classification, as well as socioeconomic indicators derived from the Mosaic classification system.

### Study population

The study cohort comprises 1,437 individuals who died between 2015 and 2023 with a registered diagnosis of ABC in the Stockholm Region, defined as a registered diagnosis of ABC (ICD-10 code C50 as a main diagnosis and a concomitant ICD 10 diagnosis of metastasis/metastases, that is, ICD 10 codes C77, C78 or C79) during the last 3 months of life, were included. Of these, 1,372/1,437 (95.5%) had distant metastases (C78, C79).

The cohort included all individuals meeting these diagnostic criteria regardless of the registered underlying cause of death. Consequently, although BC represented the primary disease driving healthcare utilization in this population, some deaths may have occurred due to acute events, treatment-related toxicity, or noncancer causes rather than progressive BC.

Both women and men were included, reflecting the population-based design of the study; however, the small number of male patients precluded sex-stratified analyses. As the analysis included the complete population meeting the inclusion criteria within the specified period, no sample size or power calculation was performed.

### Variables

Clinical variables such as frailty risk to further capture patient vulnerability and complexity was measured via the Hospital Frailty Risk Score (HFRS), which incorporates a broad range of chronic conditions and functional impairments [17–19]. Comorbidity was summarized using the Charlson Comorbidity Index (CCI), a validated composite measure in cancer and EOL research, rather than individual chronic disease categories [20, 21], as well as age, sex, socio-economic Mosaic groups, systemic cancer therapy and emergency hospital as a place of death were used to stratify risk and control for patient complexity.

Data on cancer stage and time since diagnosis were unavailable, as only the final year of life was requested from the Stockholm Regional Healthcare repository. These variables were thus excluded from the analysis.

Healthcare costs were calculated using Region Stockholm’s simulated costs model (SIM model), which distributes total regional healthcare expenditure (~SEK 82 billion in 2023) using weighted DRG diagnoses, national cost allocation codes, and actual financial data.

Cost trajectories over the final 12 months of life, disaggregated by care type (inpatient, outpatient, palliative), were analyzed in relation to demographic, socioeconomic, and clinical risk factors. Previous research indicates that frailty risk is associated with higher emergency room visits, reduced access to specialized palliative care [22], and increased costs during EOL care in both cancer [13] and nursing home populations [23] and therefore included as a variable.

### Statistical analysis

Given the non-normal, right-skewed distribution of healthcare expenditures, largely resulting from a small number of extreme-cost observations, central tendency values, medians with interquartile ranges (IQR) and means with 95% confidence intervals (CIs) were utilized to accurately summarize the data. Statistical comparisons between groups were tailored to the nature of the data. For continuous variables, nonparametric methods were applied, including the Wilcoxon rank-sum test for pairwise comparisons and the Kruskal-Wallis test for analyses involving more than two groups. Categorical variables were compared using chi-square tests. Trends in monthly cost estimates were evaluated using mean values with 95% CIs to capture variation over time. Descriptive tables were used to summarize patient characteristics without formal statistical testing, in line with STROBE recommendations.

To evaluate multivariable associations with healthcare costs, generalized linear models (GLMs) were employed, reporting rate ratios (RRs) as measures of association. High-cost users, defined as those in the top 5% of the expenditure distribution, were identified by listing frequencies. Model discrimination for logistic regression analyses was assessed using the C-statistic, which is equivalent to the area under the receiver operating characteristic (ROC) curve (AUC). Values range from 0.5 (no discrimination) to 1.0 (perfect discrimination). Analyses were performed with the aid of the SAS 9.4/Enterprise guide 8.2.

## Results

### Main study group: Costs for women with BC in ordinary accommodation

Healthcare costs were inversely proportional to age in our cohort, with the highest cost observed among individuals aged 18–69 years, who had a median cost of tSEK (thousand SEK) 576 (mean 653 tSEK). As presented in Table 1, individuals aged 70–79 years incurred a median cost of tSEK 527 (mean 587 tSEK), while those aged 80 years and above had a median cost of tSEK 413 (mean 473 tSEK). In contrast, no significant differences in costs were found among patients residing in the three socioeconomic Mosaic groups analyzed (*p* = 0.29). Similarly, the CCI did not demonstrate a significant covariation with healthcare costs (*p* = 0.6).

**Table 1:** Descriptive and clinical data in 1,437 persons with ABC, in ordinary accommodation.

Variable	Number (%)	Median cost (IQR) per individual *In thousand SEK*	Mean cost (95% CI) per individual *In thousand SEK*
**Sex, distribution**			
Women	1,426 (99)	525 (335–763)	589 (570–607)
Men	11 (1)	521 (395–791)	606 (452–761)
**Age groups, distribution**			
18–69 years	686 (48)	576 (388–812)	653 (624–682)
70–79 years	373 (26)	527 (339–761)	587 (552–621)
80 years or more	378 (26)	413 (262–647)	473 (446–501)
**Mosaic groups**			
Group 1	435 (30)	550 (354–761)	598 (567–630)
Group 2	558 (39)	518 (330–770)	583 (554–612)
Group 3	444 (31)	500 (328–764)	586 (551–622)
**CCI (*cancer excluded*)^1^**			
0	790 (55)	532 (334–743)	577 (554–600)
1 or more	647 (45)	519 (341–780)	603 (573–633)
**HFRS**			
Low risk (< 5)	859 (60)	492 (311–698)	540 (520–561)
Intermediate risk (5–15)	512 (36)	571 (364–827)	641 (609–674)
High risk (>15)	66 (4)	705 (413–1,000)	808 (673–944)
**Systemic cancer therapy**			
Yes	754 (52)	599 (438–817)	679 (653–704)
No	683 (48)	413 (255–660)	489 (565–514)
**Place of death: hospital**			
Yes	237 (17)	442 (268–738)	543 (491–596)
No	1,200 (84)	535 (356–771)	598 (578–617)

IQR: interquartile range; CI: confidence interval; CCI: Charlson Comorbidity Index; HFRS: Hospital Frailty Risk Score.

1 Kruskal-Wallis test.

2 Wilcoxon Rank Sum test.

3 Cancer diagnoses were excluded from the calculation of CCI, as all patients had cancer.

The median and mean health care expenditure costs included in the table were calculated in thousand Swedish crowns (tSEK), based on the 2023 cost level (1 Euro is approximately 11.00 SEK). The costs include all medical care costs, that is, primary care, emergency hospital care, and care at geriatric and psychiatric departments.

The HFRS was significantly associated with healthcare costs. A clear cost gradient was observed, with median expenditures increasing in proportion to frailty risk. Patients classified as having a high risk of frailty incurred the highest median costs tSEK 705 (mean 808 tSEK), followed by those at intermediate 571 tSEK (mean 641 tSEK) and low risk tSEK 492 (mean 540 tSEK), (*p* < 0.0001).

Systemic cancer therapy during the last year of life emerged as a major driver of healthcare costs. Patients who received systemic therapy had a median expenditure of tSEK 599 (mean 679 tSEK), significantly higher than the tSEK 413 (mean 489 tSEK) observed in those who did not receive such therapy (*p* < 0.0001). Finally, the place of death was also associated with differences in cost. Patients who died in emergency hospital settings incurred a median cost of tSEK 442 (mean 543 tSEK), which was lower compared to those who died in other settings: tSEK 535 (mean 598 tSEK), (*p* = 0.0004).

### Interquartile healthcare utilization and costs during the last year of life (proportion of out-patient visits, hospitalizations and specialized palliative care)

An increase in healthcare utilization was observed during the final year of life (Table 2a), paralleled by a marked rise in total healthcare costs (Table 2b). Outpatient visits remained high throughout the year, increasing from 78% of the patients with at least one visit in the first quarter (Q1) to 96% in the last quarter (Q4). However, when analyzing patients with at least one healthcare episode per quarter, the most substantial changes were observed in inpatient care (hospitalizations) and specialized palliative care (SPC), which rose from 20% and 25% in Q1 to 77% and 90% in Q4, respectively. This shift in service utilization coincided with a pronounced increase in total quarterly costs. During Q4, total costs reached 299 tSEK per patient, compared to 124 tSEK in the preceding quarter, representing a 141% increase, reflecting the higher intensity and complexity of care at EOL.

**Table 2a:** Healthcare utilization in the last year of life.

Quarters of the year	Outcare visits (%)	Hospitalization (%)	SPC (%)	Others (%)
Q1	78	20	25	78
Q2	82	24	32	78
Q3	87	33	45	79
Q4	96	77	90	75

**Table 2b:** Costs per quarter in the last year of life.

Quarters of the year	Total costs (tSEK)^1^	Outcare visits (%)	Hospitalization (%)	SPC (%)	Others (%)
Q1	74	36	26	29	9
Q2	90	32	27	33	8
Q3	124	25	30	39	6
Q4	299	10	40	48	3
Total	587				

SPC: Specialized palliative care.

1 Simulated total costs (SIM) at the year 2023 level.

The distribution of costs transitioned from predominantly outpatient-based in the early phase of the year (36% of total costs in Q1) toward inpatient and SPC services, which together accounted for nearly 90% of total costs in the last quarter. This pattern indicates a gradual reallocation of healthcare resources from routine and ambulatory management to more resource-intensive EOL and palliative care.

### Monthly progression costs

**Figure 1:**
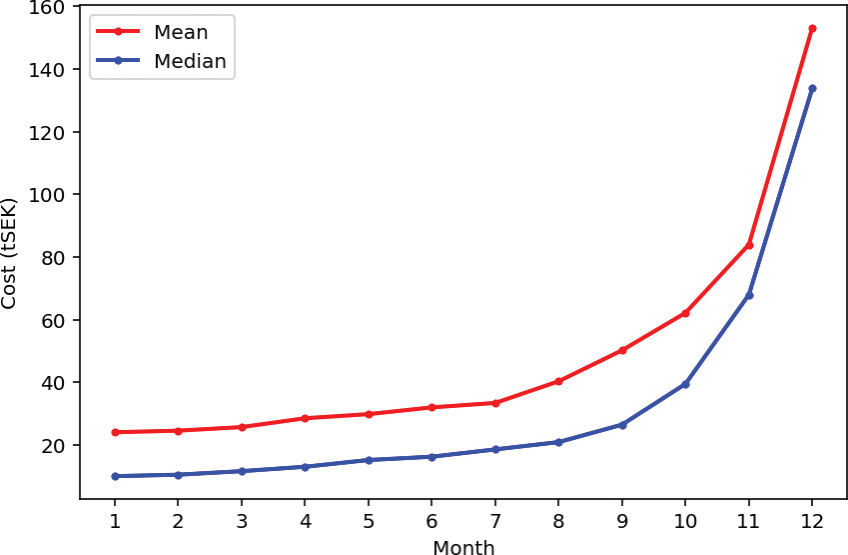
Median and mean monthly costs per individual increased progressively during last year of life, with the most pronounced rise observed during the final 3 months. (EUR 1 ≈ SEK 11). tSEK: thousand Swedish Crowns.

Both the median (IQR) and mean (95% CI) monthly costs increased progressively throughout the 12-month period. The overall rise was statistically significant for both measures, with the most pronounced escalation observed during the final months of life. Monthly comparisons demonstrated non-overlapping 95% CIs between last six consecutive months, confirming a significant upward trend, particularly during the last 3 months (Figure 1).

### Multivariable regression models for costs: GLMs

GLMs were utilized to adjust costs for relevant covariates, including age, HFRS, receipt of systemic therapy, and hospital as the place of death.

Age was a significant variable across all models, with younger patients (aged 18–69 years) and those aged 70–79 years showing higher associated costs compared to the reference group (80 years and above), as shown in Table 3. Nonetheless, the influence of age appeared attenuated in models C and D; for example, RRs for the 18–69 age group were 1.24 in model C and 1.27 in model D, where systemic therapy (model C) and both systemic therapy and hospital as the place of death (model D) were added as covariates.

**Table 3:** Generalized linear models (GLMs) estimating associations with total healthcare costs during the last 12 months of life among patients with advanced breast cancer.

Variable	Model A RR (95% CI)	Model B RR (95% CI)	Model C RR (95% CI)	Model D RR (95% CI)
Age (ref. ≥ 80 years)	1.0	1.0	1.0	1.0
18–69 years	1.38*** (1.28–1.48)	1.44*** (1.33–1.55)	1.24*** (1.15–1.34)	1.27*** (1.17–1.37)
70–79 years	1.24*** (1.14–1.35)	1.27*** (1.17–1.38)	1.16*** (1.07–1.26)	1.16*** (1.07–1.26)
HFRS (ref. HFRS < 5)		1.0	1.0	1.0
Intermediate risk (5–15)		1.22*** (1.15–1.30)	1.23*** (1.16–1.31)	1.25*** (1.17–1.33)
High risk (>15)		1.60*** (1.38–1.84)	1.67*** (1.46–1.92)	1.72*** (1.50–1.98)
Systemic tx (ref:no tx)#			1.34*** (1.25–1.42)	1.33*** (1.25–1.42)
Hospital death (ref.: not)				0.84*** (0.78–0.91)

RR: rate ratio; CI: confidence interval; HFRS: Hospital Frailty Risk Score.

Model A: Age. Model B: Age and frailty (HFRS). Model C: Age, frailty (HFRS), and systemic cancer therapies during the last year of life. Model D: Same as model C but added with hospital as place of death.

**Outcome:** Total healthcare costs per individual during the final 12 months of life, estimated using Region Stockholm’s simulated cost (SIM) model and expressed as rate ratios (RRs) with 95% confidence intervals.

*** =p-value <0.001. #Tx = therapy.

Patients classified as having a high frailty risk (HFRS > 15) consistently incurred significantly higher costs relative to the low-risk group (HFRS < 5), with RRs ranging from 1.60 to 1.72. A similar, albeit less pronounced, pattern was observed among patients in the intermediate-risk category. In a similar fashion, treatment with systemic therapy was associated with increased expenditures in the adjusted models C and D.

In contrast, patients for whom the place of death was a hospital exhibited significantly lower costs, with an RR of 0.84 (95% CI: 0.78–0.91; *p* < 0.001).

### Top 5% high-cost users

Given that a small subset of patients accounts for disproportionately high healthcare costs, we identified the top 5% of high-cost users. In this study, univariable and multivariable logistic regression analyses were conducted to further delineate the characteristics associated with membership in the top 5% of high-cost users, as detailed in Table 4.

**Table 4:** Variables associated with high-cost users, defined as the top 5% users (n = 72 of 1,437 patients).

Variable	Univariable analysis		Multivariable analysis^1^	
	OR (95% CI)	*p*-value	aOR (95% CI)	*p*-value
Age				
18–69 years	7.67 (2.75–21.36)	< 0.0001	6.47 (2.19–19.09)	0.0007
70–79 years	4.19 (1.39–12.65)	0.01	3.44 (1.10–10.72)	0.03
≥ 80 years	Ref.		Ref.	
Mosaic groups				
Groups 1+2	Ref.		Ref.	
Group 3	1.28 (0.78–2.1)	0.33	1.29 (0.77–2.15)	0.33
HFRS (ref. HFRS < 5)				
High risk (>15)	3.81 (1.67–8.69)	0.002	7.74 (3.16–18.92)	< 0.0001
Intermediate risk (5–15)	1.97 (1.19–3.25)	0.008	2.41 (1.44–4.046)	0.0008
Low risk	Ref.		Ref.	
Systemic therapy last year of life				
Yes	3.34 (1.90–5.89)	< 0.0001	2.55 (1.38–4.70)	0.003
No	Ref.		Ref.	

OR: odds ratio; CI: confidence interval; HFRS: Hospital Frailty Risk Score.

1 C-statistic was 0.73 for the final multivariable model.

Univariable analysis demonstrated that individuals in this group were more likely to be aged 18–69 years, to have a high frailty risk score (HFRS > 15), and to undergo systemic therapy. In the multivariable model, frailty risk remained a significant variable with an even stronger association, whereas the effects of age and systemic therapy were attenuated although still statistically significant.

Mosaic group classifications were not significantly associated with high-cost user status in either the univariable or multivariable models.

## Discussion and conclusion

In line with previous findings, our study confirms that healthcare costs in patients with ABC escalate significantly toward the EOL, primarily driven by increased hospitalizations, specialized palliative care, and systemic therapy intensity. The cohort included all individuals who died with a registered BC diagnosis, regardless of cause of death, reflecting heterogeneous EOL trajectories. Younger patients (aged 18–69), particularly those with good PS and high frailty risk, were strongly associated with higher total costs. While both multimorbidity and frailty, as measured by the HFRS, were significant cost drivers, frailty risk remained independently associated with elevated costs even after adjusting for age. Notably, the integration of palliative and oncologic care, especially through Sweden’s specialized home care (ASIH) model, although essential for therapy continuity, was also linked to increased EOL costs.

Healthcare costs during the final year of life are generally higher among patients with advanced-stage cancers, though the magnitude varies across diagnostic categories. For instance, hematological malignancies incur greater costs than both noncancer-related illnesses and solid tumors, as demonstrated by Strang et al. [13]. Our study focuses specifically on patients diagnosed with ABC.

We observed substantial variability in ABC-related healthcare costs, influenced by systemic therapy patterns and disease stage-specific needs, consistent with prior research [24]. Substantial healthcare use and ongoing anticancer treatment near death have also been described in studies of advanced cancer [25]. Costs rose sharply in the final months of life, echoing findings from Schneider et al., who noted similar trends driven largely by increased hospital admissions – except in certain latent subgroups whose cost trajectories diverged from the average [26].

Previous population-based studies across multiple cancer types have reported sex-related differences in EOL healthcare utilization; however, given the very small number of men in the present cohort, no conclusions regarding sex differences can be drawn from our data. Additionally, healthcare costs were significantly lower among individuals aged 70 and above compared to younger cohorts [27]. Age plays a critical role in cost analysis, potentially reflecting the willingness of younger women to pursue aggressive oncologic treatments, including advanced palliative interventions. The ongoing introduction of novel therapies (e.g. targeted agents) in both adjuvant and palliative settings further contributes to intensified healthcare consumption [28].

High-cost users represent a small subset of cancer patients who account for disproportionate share of healthcare expenditure [29]. In our cohort, individuals aged 18–69 were most likely to fall into this category, aligning with projections by Gogate et al., which estimate rising cost among younger and midlife women with metastatic BC by 2030 [30]. While comorbidities may reduce costs in early-stage cancers through earlier detection and surgical planning [31], they are associated with increased cost during the final year of life [32]. However, when age and comorbidities are considered together, the impact of comorbidities diminishes in older patients [33]. This pattern is reflected in our findings where frailty risk, rather than comorbidity, emerged as a more consistent and significant cost driver. As the effect of comorbidity on cost is obviously context dependent, high-cost use may reflect heterogeneous clinical trajectories, including acute complications such as severe infections, including septic episodes, or unexpected deaths, which cannot be distinguished in the available data.

Frailty risk, as quantified by the HFRS, emerged as a significant cost driver in our study. It is important to note that the HFRS is a proxy measure, based on 109 ICD-10 codes, capturing both frailty and multimorbidity [18]. The concept of frailty is increasingly used to guide oncologic decision-making for older patients and those nearing EOL [22, 34]. Our results align with prior studies, including Tsai et al. who found frailty to be associated with increased costs and mortality, particularly in non-metastatic settings [35].

Multiple studies have addressed the high cost of BC care at EOL, attributing it to disease progression, treatment of complications and side effects in emergency inpatient setting during the final months [4, 36]. Therapy timing is also critical: aggressive interventions near EOL may offer limited clinical benefit while increasing adverse events and inpatient care needs [37, 38]. Our findings support this evidence, showing that ABC-related costs peak in the final months, with hospitalizations and specialized palliative care rising sharply, and outpatient visits declining.

The top 5% of high-cost users in our study were younger and at higher frailty risk. In order to develop strategies toward reduction of costs, the identification of the high-cost patient population is of pivotal relevance. de Oliveira et al. found that high-cost cancer patients were more likely to have advanced-stage disease, be older, exhibit higher frailty risk, and have greater comorbidity burden, often residing in long-term care facilities [39].

High treatment intensity among younger patients with good PS is a major cost driver, even when excluding the direct cost of medications [40, 41]. Conversely, multimorbidity, such as heart failure or chronic obstructive pulmonary disease (COPD), also contributes to high costs, independent of oncologic therapy [42, 43]. Patients typically require good PS to qualify for systemic or intensive cancer therapies, excluding those with severe comorbidities [44]. Nonetheless, patients with significant multimorbidity may still incur substantial healthcare costs due to frequent medical management and hospitalizations [42, 43].

Historically, oncologic care and tumor-specific therapies have often been delivered separately from palliative care services. However, multiple studies have demonstrated that integrating palliative care with active cancer treatment can improve clinical outcomes, including therapy adherence, symptom control, and quality of life [45, 46, 47]. In Sweden, the ASIH model provides advanced palliative care at home while coordinating with hospital-based oncology services. This integrated approach is increasingly viewed as a prerequisite for enabling cancer therapies to be delivered effectively. Consequently, it is not surprising that patients who receive both oncologic and advanced palliative care exhibit higher overall healthcare costs compared to those receiving oncologic therapy alone. According to our own data, over 70% of patients were connected to ASIH in the final stages of life [20, 48].

Several limitations should be acknowledged. Without death certificates, advanced disease was defined as BC as the primary diagnosis in the last 3 months of life with distant metastases. Still, some patients may have died from acute, noncancer causes, potentially influencing cost estimates. Nevertheless, as reported in Nordic settings, registry-based palliative care data enable population-level analyses despite data limitations [49].

Our findings show that healthcare expenditures are associated with both clinical factors, including comorbidity and frailty, and demographic and systemic factors such as age, systemic therapy, and place of death. Frailty (HFRS) remained strongly associated with higher costs, supporting its value for risk stratification. Taken together, these findings identify subgroups of patients with high healthcare utilization near the EOL.

## Data Availability

The datasets generated and analyzed in this study are available upon reasonable request.
